# Developing an innovative pediatric integrated mental health care program: interdisciplinary team successes and challenges

**DOI:** 10.3389/fpsyt.2023.1252037

**Published:** 2023-11-16

**Authors:** Jason Schweitzer, Anne Bird, Hilary Bowers, Nicole Carr-Lee, Josh Gibney, Kriston Schellinger, Jasmine R. Holt, Devin P. Adams, Domonique J. Hensler, Kathryn Hollenbach

**Affiliations:** ^1^Child and Adolescent Division, Department of Psychiatry, University of California, San Diego, La Jolla, CA, United States; ^2^Transforming Mental Health Initiative, Rady Children’s Hospital-San Diego, San Diego, CA, United States; ^3^Children’s Primary Care Medical Group, San Diego, CA, United States; ^4^Department of Pediatrics, University of California, San Diego, La Jolla, CA, United States

**Keywords:** primary care, pediatrics, primary care behavioral health, integration, mental health, collaborative care, adolescents, children

## Abstract

**Introduction:**

Children and adolescents often do not receive mental healthcare when they need it. By 2021, the complex impact of the COVID-19 pandemic, structural racism, inequality in access to healthcare, and a growing shortage of mental health providers led to a national emergency in child and adolescent mental health in the United States. The need for effective, accessible treatment is more pressing than ever. Interdisciplinary, team-based pediatric integrated mental healthcare has been shown to be efficacious, accessible, and cost-effective.

**Methods:**

In response to the youth mental health crisis, Rady Children’s Hospital-San Diego’s Transforming Mental Health Initiative aimed to increase early identification of mental illness and improve access to effective treatment for children and adolescents. A stakeholder engagement process was established with affiliated pediatric clinics, community mental health organizations, and existing pediatric integrated care programs, leading to the development of the Primary Care Mental Health Integration program and drawing from established models of integrated care: Primary Care Behavioral Health and Collaborative Care.

**Results:**

As of 2023, the Primary Care Mental Health Integration program established integrated care teams in 10 primary care clinics across San Diego and Riverside counties in California. Measurement-based care has been implemented and preliminary results indicate that patient response to therapy has resulted in a 44% reduction in anxiety symptoms and a 62% decrease in depression symptoms. The program works toward fiscal sustainability via fee-for-service reimbursement and more comprehensive payor contracts. The impact on patients, primary care provider satisfaction, measurement-based care, funding strategies, as well as challenges faced and changes made will be discussed using the lens of the Reach, Effectiveness, Adoption, Implementation and Maintenance framework.

**Discussion:**

Preliminary results suggest that the Primary Care Mental Health Integration is a highly collaborative integrated care model that identifies the needs of children and adolescents and delivers brief, evidence informed treatment. The successful integration of this model into 10 primary care clinics over 3 years has laid the groundwork for future program expansion. This model of care can play a role addressing youth mental health and increasing access to care. Challenges, successes, and lessons learned will be reviewed.

## 1 Introduction

The United States (US) faces a youth mental health crisis. Rates of childhood depression and anxiety are increasing. Emergency room visits for mental health crises, suicidal ideation and attempted or completed suicides are also rising (1, 2). Although the COVID-19 pandemic played a role in exacerbating these trends (3), childhood mental health concerns were increasing even prior to 2019.

Between 2007 and 2018, the US saw a 57% increase in suicide completion among people aged 10–24 years (4). Isolation, trauma, bullying, unsafe environments, lack of adequate medical and mental health insurance, as well as relationship issues, have all been associated with adverse mental health symptoms (5, 6). Public attention concerning the negative impact of social media on mental health has been increasing (2). Although the deleterious influence of structural racism is difficult to measure, it is impossible to ignore as rates of suicide from 2003 to 2017 disproportionately increased among African American girls aged 15–17 years (6), and racial minorities continue to face significant barriers to healthcare (7–9).

As rates of mental health symptoms are increasing (10), there is greater awareness of the impact of mental health challenges and of effective interventions. The negative impact of traumatic experiences has been clearly described (11, 12), and psychotherapeutic and psychopharmacologic interventions can decrease trauma related symptoms (13). Interventions to treat anxiety and depressive symptoms are similarly effective (14, 15). Despite this knowledge, inadequate access to care continues to be persistent and widespread (2). Integrated care, or mental healthcare provided or initiated in the primary care setting, can reduce cost and improve mental health outcomes in adults (16), and bridge gaps in mental healthcare for children and adolescents.

### 1.1 Integrated care models

In a 2022 American Academy of Child and Adolescent Psychiatry (AACAP) literature review, integrated care for children and adolescents was found to be effective and work best when delivered by a multi-disciplinary team (17). According to the Substance Abuse and Mental Health Services Administration (SAMHSA) the “most collaborative” integrated care model will “function as an integrated team” at “system, team and individual levels,” follows a “shared concept of team care,” and establishes formal meeting processes with “roles and cultures that blur and blend” (SAMHSA/HRSA Center for Integrated Health Solutions, pg. 10, 2020) (18).

Primary Care Behavioral Health (PCBH) (19) and Collaborative Care (CoCM) (20, 21) are well-established integrated care models that employ high levels of collaboration. PCBH is described using the “GATHER” (Generalist, Access, Team-based, High Volume, Education and Routine) acronym (22). In PCBH, licensed mental health clinicians are embedded in the primary care office and work closely with the Primary Care Providers (PCPs). This model has been shown to be effective for children and adolescents, (23) and may reduce costs (24), but more pediatric research is needed. CoCM was initially designed to treat specific mental health conditions, such as depression, in defined patient populations, and differs from the generalist approach in PCBH that readily encourages the mental health provider to treat a range of concerns. In CoCM, a care manager (often called the Depression Care Manager or DCM) provides therapy and helps that patient navigate treatment options. A psychiatrist provides consultation and guidance to PCPs and DCMs. CoCM models often follow algorithmic approaches treatment, such that Measurement Based Care (MBC) scores prompt changes in treatment (e.g., worse scores may trigger a medication change). The Reaching out to Adolescents in Distress (ROAD) intervention (20) is an adolescent CoCM program adapted from the Improving Mood Promoting Access to Collaborative Treatment (IMPACT) model (25), that showed greater reductions in depression symptoms and rates of remission when compared to control youth.

### 1.2 The mental and physical healthcare divide

The US medical system has historically separated mental and physical healthcare (26). This division of care has resulted in mental and physical health developing into separate subsystems, each with their own unique workflow practices, attitudes, expectations and reimbursement mechanisms. Pediatric primary care providers often provide care to 20–30 patients per day, with visit lengths of 15–20 min. PCPs also provide patient advice via telephone or electronic health record (EHR) messaging and complete school, camp, government, and insurance forms. Many pediatricians do not feel they have adequate time or knowledge to treat the mental health concerns of their patients, and, as a result, refer to outside mental health providers, where timely care is often not available. Non-integrated mental health clinics and training programs do not offer learning opportunities to fully prepare for the fast pace of the primary care office. As a result many therapists do not have experience adopting a brief, efficient communication style, which may be necessary to jointly develop treatment plans with PCPs in the primary care setting. The differences between mental and physical healthcare inherent in the US provide important context for understanding the complexities of integrated care.

## 2 Methods

### 2.1 Program development

In response to the youth mental health crisis, Rady Children’s Hospital-San Diego created the Transforming Mental Health Initiative (TMH) to work toward prevention, early identification and treatment of youth mental health concerns through mental health integration, research, education, and advocacy.

In 2019, TMH convened several stakeholder meetings with local leaders and experts in community mental health, pediatrics, and integrated care to help develop plans for a significant mental health intervention. Concurrently, TMH clinical leaders completed site visits with three separate institutions with extensive integrated care experience and evaluated the rates of mental health-related emergency room visits and inpatient stays. Hospital leadership, donor relations teams, and committed donors were also engaged in the review of this information. The result of this stakeholder engagement process led TMH to propose the creation of a new Primary Care Mental Health Integration (PCMHI) program to increase early identification of mental health issues, improve access to care, and prevent the development of severe symptoms that could lead to the utilization of emergency services and significant morbidity and mortality (2).

To create PCMHI, partnerships with existing hospital-affiliated primary care practices in San Diego and Riverside counties were established, including Children’s Primary Care Medical Group (CPCMG), the second largest pediatric primary care medical group in the US. Primary care practices were identified and selected as partners based on electronic health record compatibility, existing mental healthcare infrastructure (e.g., established PHQ-9A screening at well child visits for youth aged 12 years and older), and their shared commitment to providing primary care level mental health interventions (such as PCP medication management for non-comorbid mild symptoms of depression, anxiety, or attention deficit hyperactivity disorder). Administrative leadership from TMH and the affiliated primary care sites agreed to a series of shared objectives, including: (1) increasing access to timely care; (2) using evidence-based approaches; and (3) committing to clear, open communication between PCPs and mental health providers involved in delivering integrated mental healthcare.

Team member roles (Table 1) were developed with input from primary care and mental health disciplines. PCMHI primary care partners committed to participating in ongoing continuing medical education on the management of common mental health conditions in the primary care setting. They also agreed to participate in joint decision making with patients and families on mental health treatment, offer guidance in accessing therapy, and provide psychopharmacology for mild, non-comorbid conditions.

**Table 1:** Integrated health care team member roles, Primary Care Mental Health Integration program.

Title	Role	Location	Communication	Training
Family / patient	Review and consent or opt out of program guidelines Participate in treatment planning	Primary care sites Hub sites	Typical methods EHR messaging with any team member	Psychoeducation on the nature of the program
Primary care provider	Screen for anxiety, depression Complete a warm handoff as needed Prescribe psychopharmacology for anxiety, depression and attention deficit hyperactivity disorder Seek psychiatric consultation as needed	Primary care pediatrics office	Registry conference 1x week EHR Sees patients in person or via tele-video	Integrated health topics Additional CME training
Primary care site lead	Attend weekly registry conferences Attend monthly lead meetings Foster workflow and communication	Primary care pediatrics office	Registry conference 1x week EHR	Integrated health topics Additional CME training
Therapist	Perform warm handoffs Brief Therapy – 4-6 sessions HUB therapy – 12-16 sessions	Primary care pediatrics office Hub sites	Registry conference Treatment team EHR updates for PCP following every visit Sees patients in person or via tele-video if clinically appropriate and patient preferred	Master’s level clinicians, or psychologists Weekly integrated care supervision
Psychiatrist	Provide *ad hoc* consultation Assessment and short term treatment Facilitate registry conferences Foster workflow and communication	Hub sites	Registry Conference Treatment Team EHR updates for PCP following every visit Sees patients in person or via tele-video if clinically appropriate and patient preferred	*Ad hoc* team consultation
Care coordinator	Provide referrals to families for therapy, psychiatry, community resources	Hub Sites	Registry conferences Treatment team meetings Use tele-video or telephone to communicate with families	Orientation

TMH developed a pre- and post-implementation survey to assess PCP’s beliefs and attitudes about mental health integration and the impact of PCMHI on their day-to-day patient care. Surveys were provided online to each PCP before the PCMHI program start date at their clinic. Providers received two follow up surveys at 4 months and 8 months post PCMHI program initiation.

### 2.2 Program structure

PCMHI is structured to achieve the triple aim of improved health outcomes, reduced healthcare utilization and cost, and improved patient and provider satisfaction. The program embeds therapists in partnering primary care pediatrics practices. PCMHI focuses on early identification of mental health concerns, as well as treatment for those with mild to moderate clinical presentations. It provides short-term, evidence-based treatment, working closely with primary care providers in the medical home. PCMHI also works to promote mental health screening and reduce stigma around talking about mental health in order to identify mental health challenges early, and deliver timely access to treatment to decrease symptoms and prevent clinical worsening and / or mental health crises.

PCMHI provides a continuum of care utilizing a “hub and spoke” model. “Spokes” represent the primary care pediatric sites with embedded therapists, referred to as Integrated Health Therapists (IHTs). “Hubs” represent regional, free standing mental and behavioral health clinics for higher acuity cases that may require longer term therapy or psychiatric support.

PCMHI services begin in the primary care setting where PCPs initiate referrals to the IHT if a patient or family member expresses a mental health need, the PCP is concerned there may be a mental health need contributing to a current visit, or a screening measure identifies a potential mental health concern (e.g., PHQ-9A score of 5 or higher) and the provider deems the referral appropriate. In ideal circumstances, IHTs are introduced to patients via warm handoffs from PCPs. IHTs provide brief, evidence-based therapies delivered over a series of 1–6 sessions (Table 2).

**Table 2:** Evidence informed interventions employed by integrated health therapists in the primary care mental health integration program.

Intervention	Target
Parent management training (27)	Attention deficit hyperactivity disorder
Cognitive behavioral therapy	Anxiety, depression, sleep concerns
Parent–child interaction therapy (28)	Externalizing, disruptive behaviors,
First approach skills training (FAST) (29)	Anxiety, depression, traumatic events, challenging behaviors

Hub IHTs, psychiatrists, and care coordinators work at regional Hubs. Hub IHTs provide 10–12 therapy sessions and psychiatrists offer assessment and brief treatment over a course of 4–6 sessions. Patients seen at Hub sites may receive therapy, psychiatry, or both services. Care coordinators provide community resources and referrals to PCMHI patients who require ongoing care after completing Hub treatment sessions, or who have more complex disease. Spokes are staffed with approximately one IHT and Hubs with two to four. Telemedicine can be utilized for remote care coordination, therapy and psychiatry when preferred by the patient and clinically appropriate. IHTs in both practice settings work closely with the family to better understand patient needs and to support the family.

### 2.3 PCMHI: a blended model

PCMHI blends elements of PCBH and CoCM to balance primary care needs (high volumes of patients who need to be seen quickly) and the capacities of mental health providers (who benefit from support by psychiatry to triage patients effectively, while continuing to see high volumes for brief intervention), as well as foster shared decision making for patient care. Similar to PCBH, PCMHI emphasizes a generalist approach (IHTs accept any mental health referral); access to care (IHT schedules are optimized to encourage same day introduction to PCMHI services through warm handoffs); and education (see section 3.6). PCBH elements have been essential in helping IHTs adjust to the fast pace of the primary care office and allowing PCMHI to support all patients and families in the clinic who need mental healthcare, not just specific sub-populations or patients with specific diagnoses (a potential limitation of CoCM).

PCMHI also employs several CoCM components, including routine measurement-based care (see section 3.7), weekly interdisciplinary team meetings (registry conferences) and the presence of a consulting psychiatrist on the team. These components help PCMHI engage in quality improvement through analysis of MBC data (though PCMHI does not use stepped algorithmic care *per se*), improve teamwork, and blend PCP and mental health cultures through layered communication. Presence of psychiatry also allows PCMHI to support PCPs in their medication management practice through brief psychiatric consultation, teaching or “curbside” feedback in a registry conference.

By blending the PCBH and CoCM models, PCMHI aims to provide highly collaborative, multidisciplinary team-oriented care (SAMHSA/HRSA Center for Integrated Health Solutions, 2020). Without the PCBH generalist approach and emphasis on access, PCMHI would not be able to share care for the wide range of presentations common to primary care. Without the PCBH commitment to education, PCMHI would likely not be able to maintain the workforce. Without CoCM inspired registry conferences, team communication deteriorates. Without psychiatric input and guidance, modeled after CoCM, the team’s capacity to operate at the top of their respective licenses may diminish. These elements allow PCMHI to more effectively help pediatric patients with mild to moderate mental health symptoms maintain their care within their PCP office, while still serving patients experiencing more severe symptoms, either by stabilizing them at the Hub, or bridging their care until they can be connected to higher level, specialty services outside the program. PCMHI does not provide ongoing care for clinical presentations that require specialty mental health (primary psychotic illnesses or bipolar disorder, or cases that require in-home services or partial hospital level of care), but can provide brief interventions and / or advocacy to help connect families with the services they need.

### 2.4 Interdisciplinary communication

Even with clearly defined team roles, consistent communication between PCPs and mental health providers is essential to ensure patients receive coordinated, high-quality care. Clear communication is especially important with transitions of care or in cases of atypical presentations. PCMHI has developed a range of protocols to strengthen communication.

Team communication begins with the warm handoff. The Agency for Healthcare Research and Quality defines a warm handoff as “a transfer of care between two members of the healthcare team… [that] occurs in front of the patient and family… [allowing] patients and families to hear what is said…giving them the opportunity to clarify or correct information or ask questions about their care.” (30). This interaction is hypothesized to increase engagement in subsequent services (31). Evidence on the impact of warm handoffs for mental healthcare is not entirely clear. One study found warm hand offs to be less effective for adult primary care patients whose primary language was Spanish (32). On the other hand, a systematic review of warm handoffs found increased patient engagement in services (33). The evidence specific to pediatric primary care is narrow, though Peters and colleagues found a correlation between warm handoffs and an increase from 51 to 78% in first appointment show rates (34). Another study on pediatric warm handoffs found an association with improved patient engagement, fewer no-shows and cancelations, shorter time between referral and initial scheduled mental health visit, and more total mental health visits (35).

**Figure 1:**
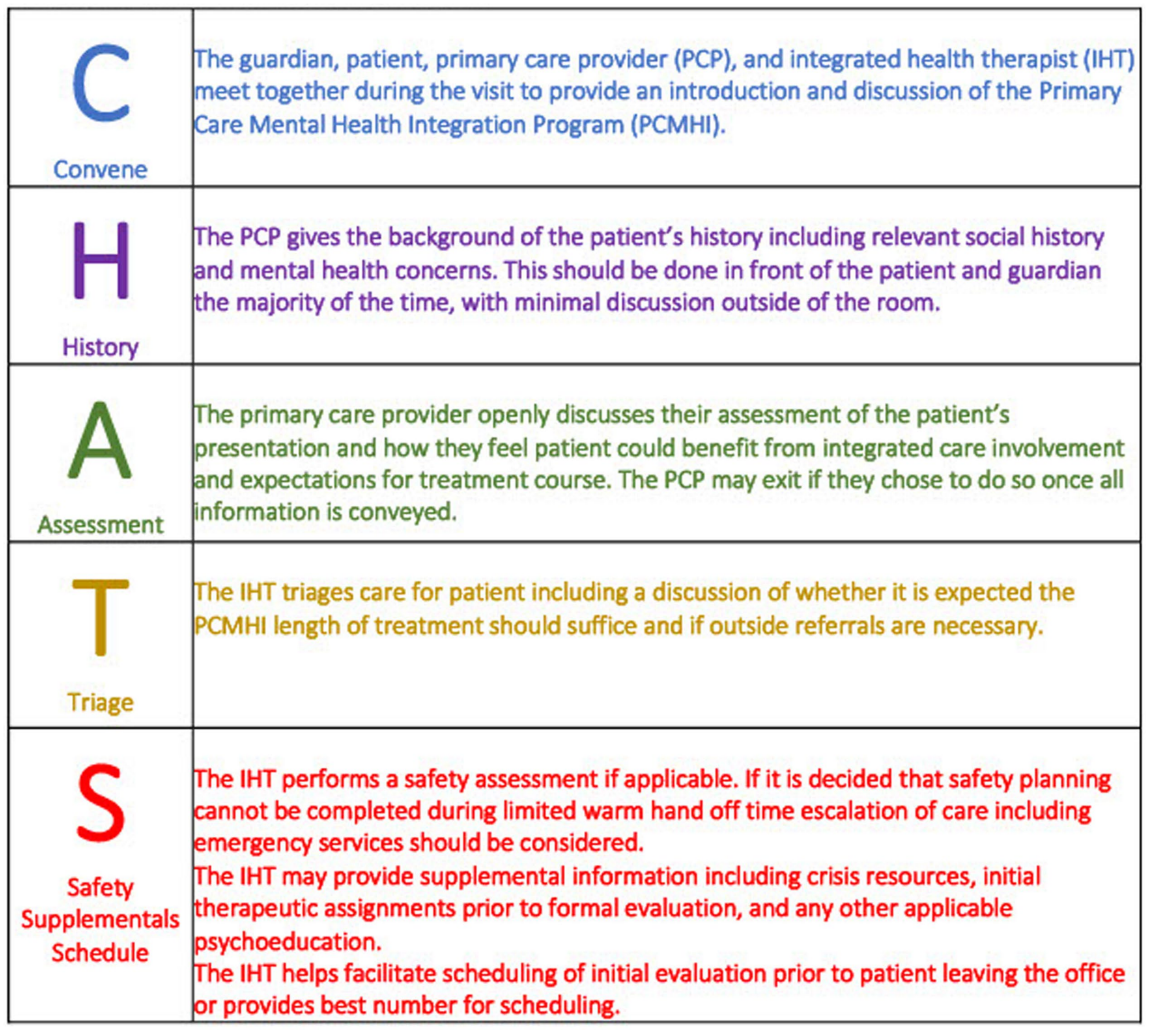
‘CHATS’ mnemonic used to describe and standardize elements of a Warm Handoffs in MHI affiliated pediatrics office.

The warm handoff leverages the PCP’s relationship with the patient and family to address mental health concerns in a trusted and judgement-free environment. PCPs participating in PCMHI are trained to follow the PCMHI-developed CHATS framework [Convene, History, Assessment, Triage, Safety (Figure 1)]. Following CHATS, the PCP, family, and IHT *convene* together in the PCP exam room to discuss, in a non-judgmental way, relevant background information and *history* for future PCMHI care. The PCP openly shares their assessment of the patient’s clinical presentation and their belief that PCMHI services may benefit the patient’s care. The PCP may then leave the exam room, allowing the IHT to further *assess* and *triage* the presenting concern and discuss treatment options, including the recommended length of care (see section 3.2). The IHT reviews program expectations and agrees upon an initial care plan with the family. The IHT may also perform a *safety* evaluation, provide supplemental information (e.g., emergency resources), and schedule an intake if indicated. Following the CHATS framework insures information is communicated in a concise, informative, and patient-centered fashion.

Additional interdisciplinary communication is formalized to maintain and encourage collaboration. Each primary care clinic identifies a PCP site lead. Site leads help facilitate communication between the PCPs at the clinic site and PCMHI clinicians. They provide real-time feedback regarding team roles and workflows and encourage their colleagues to refer eligible patients to PCMHI via the CHATS protocol. Monthly PCP site lead meetings with PCMHI leadership also serve as venue for open communication. Each primary care site holds a weekly 30–60 min registry conference, a forum for PCPs, psychiatrists, IHTs and care coordinators to discuss cases and address questions that may require input from each other’s disciplines. While not all PCPs are able to attend on a weekly basis, PCP site leads attend all registry meetings and can address or delegate questions requiring PCP input. Registry conferences also address transitions of care, which may include referrals to community services due to acuity, referrals for short-term psychiatric management, returning psychopharmacologic management to primary care. For lower acuity cases, PCPs can request real-time psychiatric feedback regarding psychopharmacologic care.

The shared EHR is used to enhance communication. IHTs use EHR messaging to provide initial clinical updates to PCPs, and over time, may use messages to coordinate treatment plans or transition care with the PCP or psychiatrist. Psychiatrists use messaging for every visit to ensure the PCP is aware of diagnostic findings or psychopharmacologic course.

### 2.5 Access to care

Same day access to mental health services is essential to the PCMHI model. When a PCP (through screeners or clinical interaction), patient or a family member identifies a mental health concern, the PCP may initiate a warm handoff. IHT schedule templates are designed to allow for warm handoffs throughout the day to insure same day access to care. Scheduling initial intakes following warm handoff visits is a priority, and the first available appointment generally occurs within 1–2 weeks due to the time needed to obtain insurance authorization.

### 2.6 Integrated care education for clinicians

Integrated care can be unfamiliar to mental health clinicians. Ongoing training and education around integrated care has helped therapist on-board and orient to PCMHI. This is accomplished through bi-weekly, CME-eligible talks on integrated health topics delivered by PCMHI clinicians to their colleagues. IHTs also meet regularly with clinical supervisors to discuss integrated care, logistical challenges, strategies for interdisciplinary communication, and clinical considerations for brief treatment. To support PCPs in their adoption of PCMHI, onboarding procedures to orient new providers to the program model have been developed. PCMHI has also partnered with an outside educational program to provide interested PCPs with a two-day in-depth training on the assessment and treatment of common mental health disorders.

For integrated care models to be sustainable and more widely adopted, PCMHI believes their benefits must be taught to future mental health providers and pediatricians. Every year, PCMHI trains 4–10 pediatric residents via a two-week integrated care elective that includes clinical observation and didactic sessions. The program also trains four child and adolescent psychiatry fellows per academic year through a year-long outpatient rotation, which includes 8 h per week of direct clinical care and 2 hours of didactic training per month. Additionally, the program recently became a training site for two psychology pre-doctoral interns.

### 2.7 Measurement based care

Measurement based care is another core component of PCMHI. TMH has been committed to using MBC data from the outset to understand the impact of PCMHI on patients and to guide the future direction of the model’s implementation. IHTs and psychiatrists complete the Clinical Global Impressions (CGI) scale for all mental health visits. Depending on the age of the patient, patient-completed standardized tools are also used, including the Patient Health Questionnaire 9 Adolescent version (PHQ-9A), Generalized Anxiety Disorder-7 (GAD-7) and Patient Symptom Checklist-17 (PSC-17). The results of these scales help guide therapeutic interventions or transitions of care and are discussed during treatment team meetings and registry conferences to support clinical decision making. Table 3. provides an overview of the MBC screening tools used by PCMHI.

**Table 3:** Patient scheduled measurement based care screening tools, primary care mental health integration program.

Tool	Definition and purpose	When
CGI	Clinical global impressions - provider perception of severity of illness and response to treatment	Every visit, all ages
GAD-7	Generalized anxiety disorder-7 - anxiety screening	Every visit, 12 years and older
PHQ-9	Patient health questionnaire, depression screening	Every visit, 12 years and older
PSC-17	Pediatric symptom checklist - 17, anxiety, depression, ADHD screening	Every Visit, 8 to 12 years of age

## 3 Results

Researchers, clinicians, and grant writers use the Reach, Effectiveness, Adoption, Implementation and Maintenance (RE-AIM) framework to evaluate the impact of behavioral health programs (36, 37). Established in 1999 (38), RE-AIM can be applied to program evaluation, grant writing and quality improvement. In the following section, multiple dimensions of PCMHI will be discussed through the lens of RE-AIM (39).

### 3.1 Reach

Since its inception in 2020, PCMHI has expanded from 1 to 10 pediatric primary care clinics and has established four Hub sites across San Diego and Riverside counties. Each clinic has unique demographics, with patient populations ranging from 2,000 to 15,000, and a distinct payor mix that includes both Medicaid (public) and commercial (private) insurances. As of 2023, PCMHI has completed 25,828 patient encounters, including 3,544 warm handoffs, 1,893 virtual triage appointments, 3,980 initial evaluations and 16,404 follow-up visits. Of the above visits, 807 were psychiatry initial evaluations and 3,824 were psychiatry follow-up appointments.

Referrals to PCMHI come directly from PCPs, and there are no fixed exclusion criteria for a referral. Each case is reviewed by a licensed clinician (IHT, clinical supervisor, or psychiatrist) and, if clinical circumstances dictate (e.g., known bipolar disorder, schizophrenia, primary substance abuse disorder), the patient may be referred to community mental health resources for specialty-level care.

### 3.2 Effectiveness

PCMHI has relied on patient volume, time to initial appointment, MBC, and provider and patient satisfaction data to measure the effectiveness of the model on access to care and patient outcomes. Clinically, MBC data indicates that patient response to therapy has resulted in a 44% reduction in anxiety symptoms, as measured by the GAD-7, and a 62% decrease in depression symptoms, as assessed by the PHQ-9A. Additional measures, including the CGI Scale for all patients and PSC-17 data for patients less than 12 years of age, have shown preliminary decreases, but the individual domain analyses have not yet been completed.

Results from pre- and post-implementation provider satisfaction surveys, which incorporated feedback from the first five clinic locations to implement PCMHI (a total of 30 PCPs), were encouraging. Prior to participating in PCMHI, many PCPs strongly disagreed that their patients had timely access to behavioral healthcare, but after program implementation, their ratings increased significantly. PCP responses also revealed a strong belief from the start in the potential of mental health integration to ease their workload and increase their capacity to treat their patients more effectively.

### 3.3 Adoption

Eleven primary care clinics were offered, and initially adopted, every aspect of the PCMHI program. Four hub sites were initiated. Since PCMHI’s start in 2020, one primary care clinic has discontinued its participation in the program (described in section 3.5).

### 3.4 Implementation

Financing integrated care comes with unique challenges. The separation of medical and mental health reimbursement has resulted in a knowledge gap vis-à-vis mental health services in health system-based and primary care settings. Freeman and colleagues argue that the financial viability of integrated care depends on specific, regional funding environments (40). They recommend several steps toward sustainability, including exploring legal requirements specific to state and clinic type, contacting payors regarding integrated care options, building a workforce with appropriate provider licenses, and establishing a business plan and capacity to audit clinical and fiscal outcomes. These recommendations influenced PCMHI’s fiscal development and have helped lay the groundwork for long-term financial sustainability.

PCMHI was started through a philanthropic donation, and is funded through a combination of philanthropy, net patient revenue and institutional support. PCMHI bills fee-for-service, and continues to seek and create opportunities for additional pay structures, including exploring value-based payments, making more effective use of available billing codes (e.g., codes for interdisciplinary meetings, para-professional services rendered, community health workers/care coordination services), and seeking grant opportunities. CoCM codes have not been used, as the structure of the registry conference has not predictably met core components for billing (41). Table 4. outlines the most billed CPT codes for PCMHI services.

**Table 4:** Most prevalent CPT codes reported by therapists, primary care mental health integration program.

PCP IHT Codes (PhD, PsyD, LCSW, LMFT, LPCC)	
Code	Code description
90791	Psychiatric diagnostic evaluation
90832	Psychotherapy, 30 min with patient
90846	Family psychotherapy (without the patient present), 50 min
90847	Family psychotherapy (conjoint psychotherapy) (with patient present), 50 min
90853	Group psychotherapy (other than of a multiple-family group)

Hub Therapy (PhD, PsyD, LCSW, LMFT, LPCC)	
Code	Code Description
90791	Psychiatric diagnostic evaluation
90834	Psychotherapy, 45 min with patient
90846	Family psychotherapy (without the patient present), 50 min
90847	Family psychotherapy (conjoint psychotherapy) (with patient present), 50 min
90853	Group psychotherapy (other than of a multiple-family group)

Psychiatry Codes (MD)	
Code	Code description
90785	Interactive complexity (List separately in addition to the code for primary procedure)
90792	Psychiatric diagnostic evaluation with medical services
90833	Psychotherapy, 30 min with patient when performed with an evaluation and management service
90836	Psychotherapy, 45 min with patient when performed with an evaluation and management service
90846	Family psychotherapy (without the patient present), 50 min
90847	Family psychotherapy (conjoint psychotherapy) (with patient present), 50 min
99205	60–74 min: Office or other outpatient visit for the evaluation and management of a new patient, which requires a medically appropriate history and/or examination and straightforward medical decision making.
99213	20–29 min: Office or other outpatient visit for the evaluation and management of an established patient, which requires a medically appropriate history and/or examination and straightforward medical decision making.
99214	30–39 min: Office or other outpatient visit for the evaluation and management of an established patient, which requires a medically appropriate history and/or examination and straightforward medical decision making.
99215	40–54 min: Office or other outpatient visit for the evaluation and management of an established patient, which requires a medically appropriate history and/or examination and straightforward medical decision making.
99358	Non-face-to-face code; Prolonged evaluation and management service before and/or after direct patient care; first hour
99451	Interprofessional telephone/Internet/electronic health record assessment and management service provided by a consultative physician, including a written report to the patient’s treating/requesting physician or other qualified health care professional, 5 min or more of medical consultative time.

There have been several adjustments to the model since inception. To increase the rates of warm handoffs, PCMHI has added PCP on-boarding trainings, increased data sharing about rates of warm handoffs, and offered yearly, site specific re-trainings on warm handoffs. To increase registry attendance, several clinics elected to alter timing or length. Educational sessions have been augmented to include CME to attract more PCPs. The MBC schedule has been altered to simplify collection (previously, MBC was offered at initial, 2 months and completion of services, but this resulted in inconsistent collection) and may continue to be adjusted, if there is evidence that the rate is too high.

### 3.5 Maintenance

As of 2023, 10 out of the 11 primary care sites that initially adopted the PCMHI program continue to offer the program. One site was discontinued as an IHT “spoke” due to a mismatch with primary care expectations around PCMHI limitations on length of care. While it is understandable that PCPs want access to longer term services for their patients, PCMHI remains committed to short-term, evidence-based interventions to increase access to timely, effective care. PCMHI relies on care coordination to connect higher acuity patients to specialty services. This highlights the challenges with maintaining model fidelity and the importance of clear communication around program expectations between PCMHI and PCP site leadership. As a result of this experience, PCMHI developed additional trainings for current and new PCP sites focused on reviewing the program model and updating integrated care teams on any changes to clinical workflows or protocols. All 10 of the current primary care sites and four Hubs remain committed to the program.

In addition to maintaining the ongoing educational efforts referenced in section 2.6, the program plans to extend additional training on First Approach Skills Training (FAST) (Table 1) to its IHTs.

Additional efforts to maintain PCMHI include plans for more extensive analysis of MBC data to include patient level sub-group differences in treatment completion and symptom response and more frequent report outs to clinicians on trends in PHQ-9A, GAD-7, PSC-17 and CGI data. Future areas of investigation include comparing PCMHI to treatment as usual of mental health issues in pediatric primary care, measuring the impact of warm handoffs on no show and cancelation rates, and exploring the impact of training and education on providers and trainees from all disciplines.

## 4 Discussion

PCMHI evolved during a national crisis in child and adolescent mental health. The program was created in response to a need for more accessible and effective youth mental health services. It drew from PCBH and CoCM to balance primary care and mental health provider needs, while simultaneously encouraging shared decision-making in patient care. In the 3 years since its creation, PCMHI has made headway establishing a model that can play a role in addressing youth mental health needs, but the road to change is never easy. Reflecting on the program’s development, implementation, and maintenance through the RE-AIM framework has allowed for a review of the successes and challenges associated with the model’s implementation and an opportunity to share key lessons learned.

Since 2020, PCMHI has completed over 25,000 patient visits across its primary care and Hub sites and has maintained consistent, timely access to care for patients. Preliminary analysis of MBC data shows the model is effective, as patients have reduced anxiety and depression symptoms as measured by changes in GAD-7 and PHQ-9A screenings. Furthermore, PCP attitudes toward PCMHI are positive, as they believe the model increases access to effective mental healthcare for their patients while easing their workload. In addition to the committed and compassionate hard work of all PCPs, mental health providers, and staff involved in PCMHI, the maintenance of clear, ongoing communication across disciplines and focus on continued quality improvement is essential to the success of the program.

As shared in section 3.4, the implementation of integrated care models is fiscally difficult and this proved to be the case for PCMHI. As of 2023, the program relies on fee-for-service reimbursement as well as continued institutional and philanthropic support, while actively working toward long-term fiscal sustainability via additional revenue sources, including more comprehensive payor contracts.

It is PCMHI’s hope that, by sharing this experience, it can help other systems of care realize the potential for positive change through integrated care for children and adolescents. Recognizing that PCMHI is still in its early years and is a work in progress, it will continue to analyze all aspects of the program and adapt as needed.

## Data availability statement

The original contributions presented in the study are included in the article/Supplementary material, further inquiries can be directed to the corresponding author/s.

## Author contributions

JS and KH contributed to the conception and design of the paper. JS wrote the first draft of the manuscript and first draft of revision. KH provided the extensive guidance for the manuscript. JH and NC-L wrote sections of the manuscript and DH, HB, JG, KS, DA, and AB contributed to the sections of the manuscript. JG wrote and designed (Figure 1). DA provided the current patient profile data and data for statistical analysis. KH performed the statistical comparisons for patient screening data. All authors contributed to manuscript revisions and read and approved the submitted version.
