# Supplementary material for: Developing an innovative pediatric integrated mental health care program: interdisciplinary team successes and challenges
Source: Front Psychiatry. 2023 Nov 16;14:1252037. doi: 10.3389/fpsyt.2023.1252037 (PMC10693412; doi:10.3389/fpsyt.2023.1252037)
Supplement: Supplementary file 1 [file Data_Sheet_1.docx]

Supplementary Material

**Developing an Innovative Pediatric Integrated Care Program: Interdisciplinary Team Successes and Challenges**

**Jason Schweitzer, MD^* 1, 2^, Anne Bird, MD^1, 2^, Hilary Bowers, MD^1, 2, 3^, Nicole Carr-Lee, PsyD^2^, Josh Gibney, MD^1, 2^, Kriston Schellinger, PhD^2^, Jasmine R. Holt, PsyD^2^,**

**Devin P. Adams, MPH^2^, Domonique J Hensler, MHA^2^, Kathryn Hollenbach, PhD^2, 4^**

1 Department of Psychiatry, Child and Adolescent Division, University of California San Diego, La Jolla, CA, USA

2 Transforming Mental Health Initiative, Rady Children’s Hospital-San Diego, San Diego, CA, USA

3 Children’s Primary Care Medical Group, San Diego, CA, USA

4 Department of Pediatrics, University of California San Diego, La Jolla CA, USA

*** Correspondence:**  
Jason Schweitzer, MD

[jaschweitzer@health.ucsd.edu](mailto:jaschweitzer@health.ucsd.edu)

# Supplementary Data

# NA

# Supplementary Figures and Tables

## Supplementary Figures

Supplementary Figure 1. ‘CHATS’ mnemonic used to describe and standardize elements of a Warm Handoffs in the Primary Care Mental Health Integration program.

| **C**  **Convene** | **The guardian, patient, primary care provider (PCP), and integrated health therapist (IHT) meet together during the visit to provide an introduction and discussion of the Primary Care Mental Health Integration Program (PCMHI).** |
| --- | --- |
| **H**  **History** | **The PCP gives the background of the patient’s history including relevant social history and mental health concerns. This should be done in front of the patient and guardian the majority of the time, with minimal discussion outside of the room.** |
| **A**  **Assessment** | **The primary care provider openly discusses their assessment of the patient’s presentation and how they feel patient could benefit from integrated care involvement and expectations for treatment course. The PCP may exit if they chose to do so once all information is conveyed.** |
| **T**  **Triage** | **The IHT triages care for patient including a discussion of whether it is expected the PCMHI length of treatment should suffice and if outside referrals are necessary.** |
| **S**  **Safety**  **Supplementals**  **Schedule** | **The IHT performs a safety assessment if applicable. If it is decided that safety planning cannot be completed during limited warm hand off time escalation of care including emergency services should be considered.**  **The IHT may provide supplemental information including crisis resources, initial therapeutic assignments prior to formal evaluation, and any other applicable psychoeducation.**  **The IHT helps facilitate scheduling of initial evaluation prior to patient leaving the office or provides best number for scheduling.** |
